# Elevated LINC00909 Promotes Tumor Progression of Ovarian Cancer via Regulating the miR-23b-3p/MRC2 Axis

**DOI:** 10.1155/2021/5574130

**Published:** 2021-07-21

**Authors:** Xu Yang, Guixia Wu, Fan Yang, Lang He, Xiaohui Xie, Lan Li, Lei Yang, Yulan Ma, Qin Zhang, Jiao Chen, SiYing Zou, Qian Han, Yan Wang, Shuai Liu, Juan Li, Bin Han, KaiJiang Liu

**Affiliations:** ^1^Department of Obstetrics and Gynecology, The Fifth Affiliated People's Hospital of Chengdu University of Traditional Chinese Medicine, No. 33, Mashi Street, Wenjiang District, Chengdu 610000, China; ^2^Department of Gynecological Oncology, Ren Ji Hospital, School of Medicine, Shanghai JiaoTong University, No. 145 Middle Shandong Road, Huangpu District, Shanghai 200001, China; ^3^Department of Physiology, School of Basic Medicine, Xinjiang Medical University, China; ^4^Department of Pathology, The Fifth Affiliated People's Hospital of Chengdu University of Traditional Chinese Medicine, Chengdu, Sichuan, China; ^5^Department of Oncology, The Fifth Affiliated People's Hospital of Chengdu University of Traditional Chinese Medicine, Chengdu, Sichuan, China; ^6^Department of Gynecology and Obstetrics, Guangzhou Women and Children's Medical Center, Guangzhou Medical University, Guangzhou, China; ^7^Department of Laboratory, The Fifth Affiliated People's Hospital of Chengdu University of Traditional Chinese Medicine, Chengdu, Sichuan, China

## Abstract

Ovarian cancer (OC), the third common gynecologic malignancy, contributes to the most cancer-caused mortality in women. However, 70% of patients with OC are diagnosed at an advanced stage, of which the 5-year survival is less than 30%. Long noncoding RNAs (long ncRNAs or lncRNA), a type of RNA with exceeding 200 nucleotides in length but no protein-coding capability, have been demonstrated to involve the pathogenesis of various cancers and show considerable potential in the diagnosis of OC. In this study, we found that the LINC00909 expression in tumor and serum specimens of OC patients was elevated, determined by real-time quantitative, and droplet digital PCR. In receiver operating characteristic (ROC) analysis, our results revealed that serum LINC00909 distinguished cancers from normal ovarian tissue with 87.8% of sensitivity and 69.6% of specificity (AUC, 81.2%) and distinguished serous ovarian cancer from normal ovarian tissue with 90.0% of sensitivity and 75.9% of specificity (AUC, 84.5%). Furthermore, we observed that the tumor and serum LINC00909 level was positively associated with the International Federation of Gynecology and Obstetrics (FIGO) stage and the Eastern Cooperative Oncology Group (ECOG) score (reflecting patients' performance status). Also, patients with low serum LINC00909 level showed a longer overall (hazard ratio, HR = 1.874, *p* = 0.0004) and progression-free (HR = 1.656, *p* = 0.0017) survival. Functional assays indicated that the elevation of LINC00909 expression contributes to cell proliferation, migration, and invasion capability of ovarian cancer cells. Besides, we demonstrated that LINC00909 functions as a competing endogenous RNA (ceRNA) of MRC2 mRNA by sponging miR-23-3p, and thereby promotes epithelial-to-mesenchymal transition (EMT) of ovarian cancer cells. Therefore, we highlight that the LINC00909/miR-23b-3p/MRC2 axis is implicated in the pathogenesis of ovarian cancer, and serum LINC00909 may be a promising biomarker for the diagnosis of OC.

## 1. Introduction

Ovarian cancer (OC), the third common gynecologic malignancy, contributes to the most cancer-caused mortality in women and increases with approximately 22,000 new cases and 14,180 deaths every year in the United States [1, 2]. In histological subtypes, approximately 90% of OC is epithelial ovarian cancer, which consists of serous, endometrioid, and clear-cell ovarian carcinoma [2–4]. In the examination, the early-stage OC, such as the International Federation of Gynecology and Obstetrics (FIGO) stage-I/II, is difficult to diagnose because most symptoms of OC are nonspecific. For example, symptoms of early OC may appear similar to irritable bowel syndrome [5]. Due to the lack of early detection or screening test, about 70% of patients with OC are diagnosed at an advanced stage (FIGO stage III/IV), a mere 30% of which are expected to survive 5 years [1, 6, 7]. In contrast, the 5-year overall survival of patients with early-stage OC was reported to be 70-95% [3, 4]. Therefore, it is definitely beneficial to reducing OC-caused mortality to develop more effective diagnostic methods for this disease.

tLong noncoding RNAs (long ncRNAs or lncRNAs) are a type of RNA with lengths exceeding 200 nucleotides but have no protein-coding capability [8–13]. Based on their genomic location, lncRNAs are categorized as intergenic, intronic, divergent, and antisense subtypes, especially the long intervening/intergenic noncoding RNAs (lincRNAs) [10, 14–20]. In functions, convincible studies by independent groups have shown that lncRNAs are implicated in a variety of cancers (such as cervical, bladder, colon, lung, glioblastoma, hepatocellular, and prostate) by influencing cell proliferation, differentiation, apoptosis, etc. [12, 13, 21–27]. By analyzing the OC transcriptome datasets from TCGA, Liang et al. found that PTAR, a lncRNA, was significantly upregulated in the mesenchymal subtype samples compared with the epithelial subtype samples [28]. In 2017, Wu et al. demonstrated that ABHD11-AS1 expression in epithelial ovarian cancer tissues was higher than that in normal ovarian tissue and was positively associated with the tumor stage (stage I/II vs. stage III/IV) [29]. Moreover, Wang et al. proved that, compared with cisplatin-sensitive ovarian cancer tissues and cells, PANDAR exhibits higher expression in cisplatin-resistant ovarian ones, and loss-function of PANDAR causes a significant tumor growth arrest [22]. Accordingly, lncRNAs exhibit a huge potential in the clinical application of OC diagnoses, such as tumor grading and chemosensitivity detection.

In this study, by reanalyzing the published OC transcriptome microarray datasheet in GEO, we found an upregulated lncRNA, LINC00909, both in low- and high-grade serous ovarian cancers. This finding was further validated in tumor and serum specimens of 217 OC patients by comparing them with 89 normal ovarian tissues. Moreover, we comprehensively evaluated the advantages of the tumor and serum LINC00909 in possible clinical applications including tumor diagnosis and grading and prognosis prediction. In a mechanism, we demonstrated that the elevated LINC00909 level in ovarian cancer cells upregulates the expression of MRC2 by sponging miR-23b-3p and thereby promotes cell proliferation, migration, invasion, and EMT of ovarian cancer cells.

## 2. Methods and Materials

### 2.1. Patients

In the present study, a total of 89 normal participants and 217 patients with histologically validated primary ovarian cancer were enrolled in The Fifth Affiliated People's Hospital of Chengdu University of Traditional Chinese Medicine from Jan 2014 to Apr 2019. The specimens of primary ovarian cancer and the related blood were collected and stored at -80°C for a long time. The tumor sections of each patient were checked by 3 oncologists. The clinical manifestations of patients are presented in Table 1. All the tumors were graded by 3 independent pathologists using the International Federation of Gynecology and Obstetrics (FIGO) grading system (good to bad: I-IV) [5]. The performance status of patients was assessed by oncology healthcare professionals using the Eastern Cooperative Oncology Group (ECOG) score (good to bad: 0-5) [30]. All participants or their families signed the written informed consent. The methodologies involved in this study conformed to the Declaration of Helsinki. The protocols used in the present study also were approved by the ethics committee of Chengdu University of Traditional Chinese Medicine.

### 2.2. RNA Extraction

Tumor tissues were immediately immersed into RNAlater RNA Stabilization Reagent from Thermo Fisher Scientific (Cat: AM7021, Waltham, USA) at 4°C for 24 hrs and then stored at -80°C for a long time. Blood samples in a vacuum collection tube were incubated in 37°C water bath for more than 30 min to completely coagulate. Then, the blood samples were centrifugated for 10 min at 1500 g (room temperature) to obtain cell-free serum fraction, which was immediately frozen at -80°C for further use. The RNA of tumor tissues was extracted by RNeasy Protect Mini Kit from Qiagen (Cat: 74124, Germantown, MD, USA), and serum RNA by QIAamp UltraSens Virus Kit from Qiagen (Cat: 53704, Germantown, MD, USA), following the manufacturer's instruction.

### 2.3. Real-Time Quantitative and Droplet Digital PCR

For the quantification of tumor tissue RNA, real-time qPCR was performed on 7500 Real-Time PCR systems (Applied Biosystems, Waltham, USA) using TB Green Fast qPCR Mix from TaKaRa (Cat: RR430A, Beijing, China). The relative level of targets was analyzed by the *ΔΔ*Ct method using GAPDH mRNA as the internal control. In the case of serum RNA, droplet digital PCR was carried out for absolute quantification using droplet digital polymerase chain reaction (ddPCR) Supermix on a BioRad QX200 Droplet Digital PCR System (Munich, Germany) following the manufacturer's instruction. Briefly, 20 *μ*l PCR was mixed with 70 *μ*l droplet generator oil pipetted into the cavities of the droplet generator cartridges (Bio-Rad) to generate droplet, and then, 40 *μ*l droplet suspension was transferred into 200 *μ*l PCR tubes. After a brief spin down, the tubes were located into the PCR machine to perform the program of 95°C for 10 min followed by 41 × 95°C for 30 s and 60°C for 60 s and 98°C for 10 min. The level of each target was detected using a QX200 droplet reader, and the data analysis was analyzed using the QUANTALIFE (Bio-Rad) software. All the involved primers for PCR were listed in Table 2.

### 2.4. Cell Culture

SKOV3 (Cat: HTB-7) cells were purchased from American Type Culture Collection (ATCC) and cultured in ATCC-formulated McCoy's 5a Medium Modified (Cat: 30-2007) plus 10% fetal bovine serum (FBS, Gibco, Rockville, USA) and 50 *μ*g/ml penicillin/streptomycin (P/S, Gibco, Rockville, USA). CAOV4 (Cat: HTB-76) cells also were purchased from ATCC but cultured in ATCC-formulated Leibovitz's L-15 Medium (Cat: 30-2008) plus 10% FBS and 50 *μ*g/ml P/S. A2780 (Cat: 93112519) cells were purchased from the European Collection of Authenticated Cell Cultures (ECACC) and cultured in RPMI-1640 plus 2 mM Glutamine, 10% FBS, and 50 *μ*g/ml P/S. JHOS4 (Cat: RCB1678) cells were purchased from cell bank of RIKEN BioResource Research Center and cultured in DMEM/HamF12 plus 0.1 mM NEAA, 10% FBS, and 50 *μ*g/ml P/S. EFO21 (Cat: ACC235) was purchased from DSMZ-German Collection of Microorganisms and Cell Cultures and cultured in RPMI-1640 plus 10% FBS, 50 *μ*g/ml P/S, 2 mM glutamine, 1 × MEM nonessential amino acids, and 1 mM sodium pyruvate. OVCAR4 cells were purchased from COBIOER Company (Nanjing, China) and cultured in RPMI-1640 supplemented with 10% FBS plus 50 *μ*g/ml P/S. All the cells were maintained in a CO_2_ incubator (with inner 37°C).

### 2.5. Generation of Stable Cell Lines

For the generation of stable overexpression and knockdown SKOV3/OVCAR4 cell lines, we utilized pLVX (Cat: #125839, Addgene) and pLKO.1 (Cat: #8453, Addgene) lentivirus vector to deliver gene sequences, respectively, and psPAX2 (Cat: #12260, Addgene) and pMD2.G (Cat: #12259, Addgene) auxiliary constructs for lentivirus packaging. Firstly, HEK 293T (1 × 10^6^ cells) were cotransfected with the constructs mentioned above using PolyJet (SL100688, Signagen, USA). 8 hrs after transfection, the medium of 293T cells was refreshed with a complete culture medium containing 10% FBS plus 50 *μ*g/ml P/S. 48 hrs later, the supernatants were collected for virus particle purification using Lentivirus Concentration Reagent (Cat: BW-V2001-03, Chuangwei biosciences, China). After the determination of virus titer, the virus particle and 10 *μ*g/ml polybrene (Cat: TR-1003-G, Sigma, USA) were added to infect OVCAR4 and SKOV3 cells. 2 days after infection, cells were added with 2 *μ*g/ml puromycin (Cat: A1113802, ThermoFisher Scientific, USA) for the selection of virus-infected cells. Before usage for further experiments, all the cell lines were subjected to real-time qPCR to determine the expression level of target genes. The shRNA sequences involved in the study were listed in Table 2.

### 2.6. MTT Assay

SKOV3/OVCAR4 cell lines (2 × 10^4^ cells/ml) were subsequently seeded into a 96-well with an interval of 0.5 days. After the growth of 2-3 days, cells in each well were incubated with MTT (3-(4, 5-dimethylthiazol-2-yl)-2,5-diphenyltetrazolium bromide) solution (20 *μ*l). After incubation at 3°C for 4 hrs, cells were supplemented with 150 *μ*l DMSO to dissolve the obtained crystal at room temperature for 15 min. Finally, the cell growth curve was determined by detecting optical density (OD) at a wavelength of 450 nm using a Multiskan SkyHigh analyzer (Thermo Fisher, USA).

### 2.7. Wound Healing Assay

SKOV3 and VOCAR4 cell lines were seeded in a 12-well plate (2.5 × 10^5^ cells/well). The next day, the confluent cell monolayer was scratched to create an artificial wound using a 10 *μ*l pipette tip. On day 1, 2, and 3 after the scratch, the wound width was recorded using an inverted microscope.

### 2.8. Transwell Assay

SKOV3 and VOCAR4 cell lines were seeded on the upper side of the Matrigel-coated transwell chamber with a cell density of 3 × 10^4^ cells/well. The medium of the bottom side was supplied with 20% FBS. After the growth of 24 hrs, the cells of the bottom side were fixed using the 4% paraformaldehyde for 30 min and immediately followed by stained using crystal violet for 30 min. Finally, the migrated cells to the bottom were to be imaged using a microscope for cell number counting.

### 2.9. Western Blot

The total protein of SKOV3/OVCAR4 cells was extracted using radioimmunoprecipitation assay buffer (Beyotime, Cat: P0013C). After quantification of total protein concentration, each sample was separated by electrophoresis of 10% sodium dodecyl sulfate- (SDS-) polyacrylamide gel (Beyotime, Cat: P0509S) and then transferred to a nitrocellulose filter membrane (Whatman, Cat: 10600001). After blocking using 5% fat-free milk at room temperature for 1 hr, the membranes were probed using primary antibodies at 4°C overnight. The next day, the membranes were briefly washed with 1 × Tris-buffered saline containing Tween-20 (TBST, Beyotime, Cat: ST677) for 3 × 5 min, followed by being incubated with secondary antibodies (1 : 5000) for 2 hrs at room temperature. Finally, the protein bands were visualized using High-sig ECL Western Blotting Substrate (Tanon, Cat: 180-501) following the manufacturer's instructions The protein bands were quantified using the software of ImageJ. Anti-MRC2 (Cat: ab70132) was purchased from Abcam, and E-cadherin (Cat: #14472), N-cadherin (Cat: #13116), and vimentin (Cat: #5741) from Cell Signaling Technology (CST).

### 2.10. Dual-Luciferase Reporter Assay

**Figure 1:**
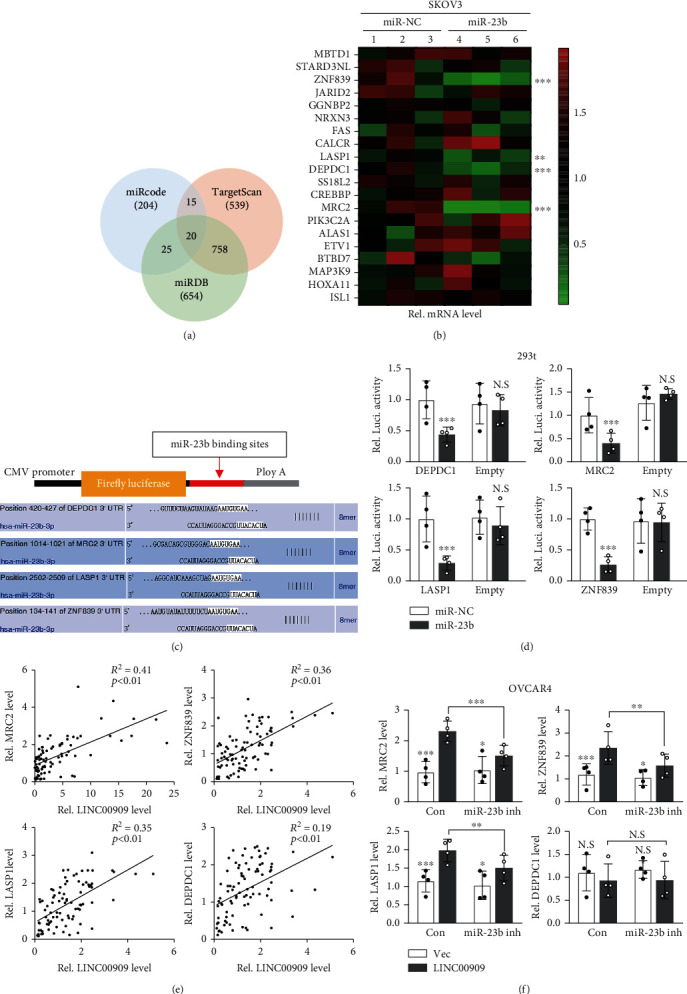
LINC00909 serves as a ceRNA of MRC2, LASP1 and ZNF839 by sponging miR-23b (a) Venn diagram showed the overlap of the miR-23b-3p's targets from miRcode, TargetScan and miRDB database. (b) The levels of putative targets of miR-23b-3p in SKOV3 were determined by real-time qPCR. miR-23b-3p was stably transfected into SKOV3 cells. (c-d) Dual-luciferase reporter assay verified the interaction between miR-23b-3p and its putative mRNA targets. C shows the sequences of binding sites of miR-23b-3p and its putative targets. 293T cells were transiently cotransfected with the indicated constructs. (e) Scatter plot showed the correlation between LINC00909 and MRC2/ZNF839/DEPC1/LASP1 mRNA in clinical specimens. *R*: Pearson correlation coefficient. (f) Real-time qPCR revealed mRNA level of MRC2/ZNF839/DEPC1/LASP1 in OVCAR4 cells. In OVCAR4 cells (in S4C), LINC00909 was stably transfected, and miR-23b-3p inhibitor was transiently transfected.

To prove the interaction between LINC00909 and its putative miRNA targets by ENCORE online tools [31] (Stable1), we performed a dual-luciferase reporter assay using the LINC00909 Firefly luciferase construct as in Figure 1(a) and Renilla luciferase construct. Also, the direct interaction between miR-23b-3p and DEPDC1/MRC2/LASP1/ZNF839 mRNA was validated by dual-luciferase reporter assay, in which the Firefly luciferase constructs were shown in Figure 1(c).

12 hrs before transfection, 293T cells (0.2 × 10^6^) were seeded into 12-well plates. The cells with 80% confluence were transiently cotransfected with Firefly (empty of sequence-specific), Renilla luciferase constructs, and miRNA-mimics or miRNA-NC (negative control). 48 hrs after transfection, cells were harvested for the luciferase activity determination using a Dual-Luciferase Assay kit (Promega, Cat: E1910) following the manufacturer's protocol. The Firefly luciferase activities were normalized by Renilla luciferase. Each sample in the dual-luciferase reporter assay was assayed four times.

### 2.11. Data Mining

The published transcriptomic data of serous ovarian cancer (both high and low grade, 6 duplications for each group) and normal fallopian tube (control group, 6 duplications) from Gene Expression Omnibus (GEO, GSE135886) was used for exploring the roles of lncRNAs in OC diagnosis. The miRNA expression data were analyzed using the datasheet of GSM4797395 (GEO). We took advantage of R language packages including GEOquery, Limma, and UMAP for finding the differential expressed genes, in which *p* value < 0.01 and fold change > 2 were selected as the cutoff. The expression and subcellular location information of LINC00909 in cell lines were analyzed using database LncATLAS (https://lncatlas.crg.eu).

### 2.12. Statistical Analysis

In this study, the software of GraphPad prism 7 (CA, USA) was used for statistical analysis. In the comparison of lncRNAs in each group, a nonparametric test based on the Kruskal-Wallis test was used, followed by Dunn's multiple comparison correction. Receiver operating characteristic (ROC) curve analysis was carried out to estimate the diagnostic performance of lncRNAs using the pROC package. Spearman's rank correlation test was carried out to evaluate the correlation between tumor and serum lncRNAs. Also, Kaplan–Meier analysis and the log-rank test were performed for progression-free survival (PFS) and overall survival (OS). For two-group comparison, student's *t*-test or Mann–Whitney test (if the variances of samples are heterogeneous) was used; for comparison with more than three groups, one-way ANOVA followed by post hoc Tukey's multiple comparisons was used. A *p* value < 0.05 was considered statistical significant (N.S, no significance; ^∗^*p* < 0.05, ^∗∗^*p* < 0.01, and ^∗∗∗^*p* < 0.001).

## 3. Results

### 3.1. LncRNAs Are Differentially Expressed in OC Compared with Normal Ovarian Tissues

**Figure 2:**
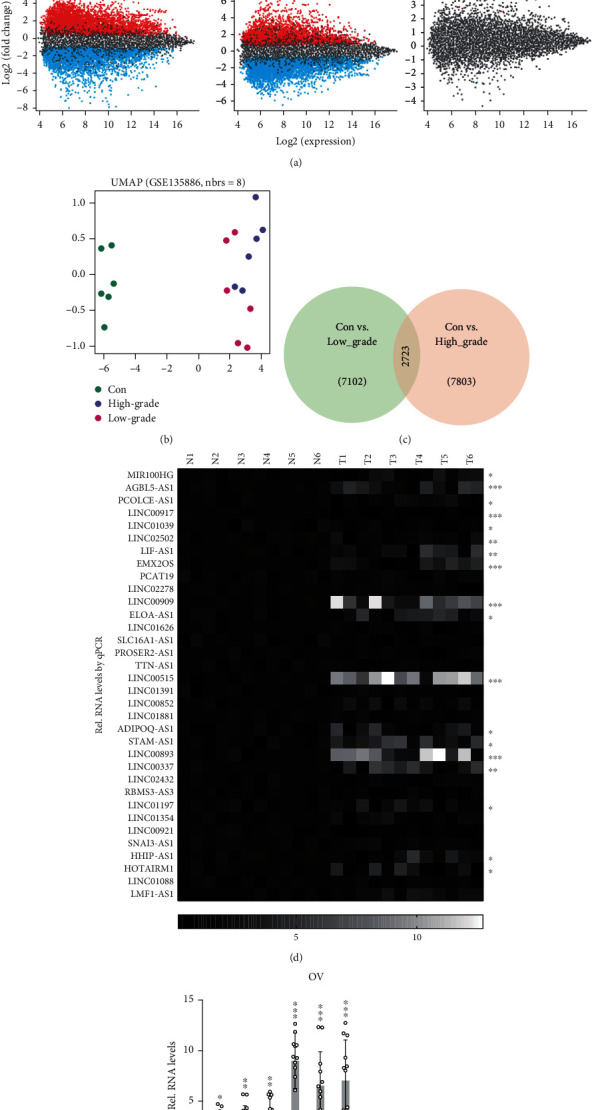
LncRNAs are differentially expressed in OC compared with normal ovarian tissues. (a) Volcano and Meandiff plot of each two groups from the GSE135886 datasheet. Con,: normal fallopian tube tissue; low: low-grade primary ovarian cancer; high: high-grade primary ovarian cancer; fold change > 2 and *p* value < 0.01 serve as the cutoff. (b) Multidimensional scaling of three groups in (a). (c) Venn graph shows the overlap of differentially expressed probes between the indicated two groups. The groups are the same as in (a). (d, e) The level of lncRNAs was determined by real-time qPCR. 6 paired specimens of normal and ovarian cancer tissues were used for lncRNA relative quantification. N: normal tissues; T: tumor tissues.

To explore the diagnostic significance of lncRNAs in the patients with primary ovarian cancer, we identified the differentially expressed genes (DEGs) in ovarian cancer (both low and high grade) compared with normal fallopian tube tissues using the published microarray data in the GEO database. We found 7102 differentially expressed probes in comparison to normal fallopian tube vs. low-grade ovarian cancer, as well as 7803 ones in comparison to normal fallopian tube vs. high-grade ovarian cancer (Figures 2(a) and 2(c)). In multidimensional scaling, the low- and high-grade groups show a close distance with each other (Figure 2(b)), suggesting there is considerable overlap in their differential expressed genes. Indeed, these two groups share 2723 differentially expressed probes (annotated as 2433 genes), including 2246 protein-coding genes, 37 lincRNAs, and 31 antisense transcripts.

Furthermore, the expression of all the identified differentially expressed lincRNAs was determined in 6 paired specimens of ovarian cancer and adjacent normal tissues by real-time qPCR. Fortunately, our results showed that lincRNAs levels, such as LINC00893, LINC00921, AGBL5-AS1, LIF-AS1, EMX2OS, LINC00909, ELOA-AS1, and LINC00515, were significantly elevated in tumors, and in contrast, LINC00917, LINC00921, etc. were downregulated (Figures 2(d) and 2(e)).

### 3.2. LINC00909 Expression Is Elevated in Tumors and Serum of OC Patients

**Figure 3:**
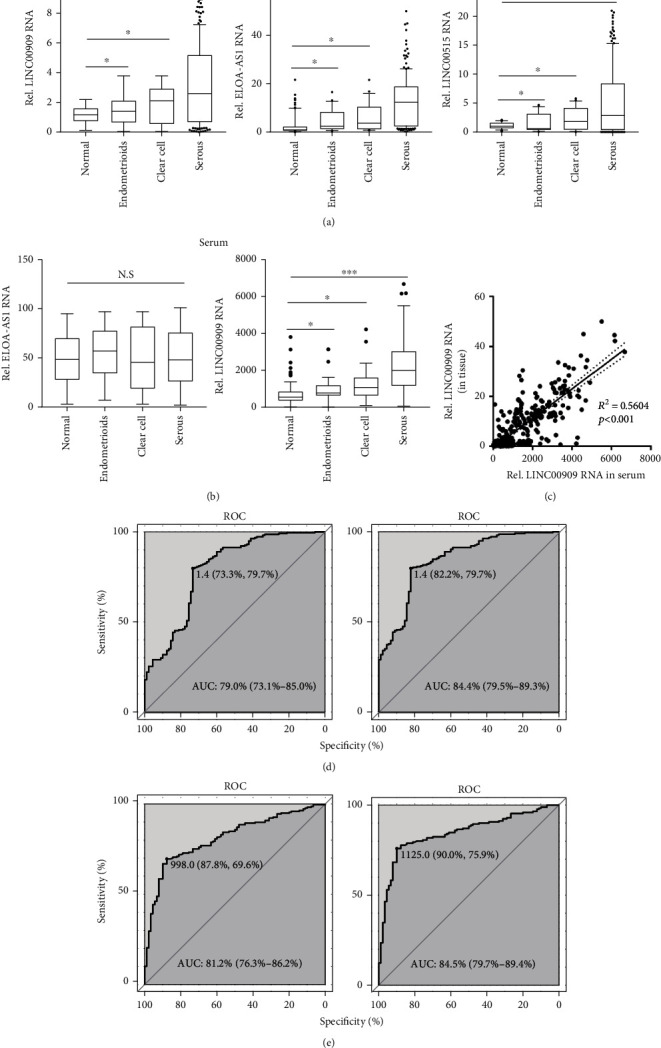
LINC00909 expression is elevated in tumors and serum of OC patients. (a) The level of lncRNAs was determined by real-time qPCR. Tissues of normal ovarian (*N* = 89), serous (*N* = 170), endometrioid (*N* = 25), and clear-cell ovarian cancer (*N* = 22) were used for lncRNA relative quantification. (b) The level of lncRNAs was determined by droplet digital PCR. Tissues of normal ovarian (*N* = 89), serous (*N* = 170), endometrioid (*N* = 25), and clear-cell ovarian cancer (*N* = 22) were used for lncRNA quantification. (c) Scatter plot of tissue and serum LINC00909 level. The expression level of LINC00909 is from (a) and (b). *R*: Pearson correlation coefficient. *N* = 306. (d, e)Tissue (d) and serum (e) LINC00909-based ROC analysis for OC diagnosis. AUC: area under curve. *N* = 306.

Next, we further validated the identified lncRNAs in 89 normal ovarian tissues and 217 primary ovarian tumors (including 170 serous, 25 endometrioid, and 22 clear-cell ovarian cancers). Our results revealed that LINC00909, ELOA-AS1, and LINC00515 expressions were obviously increased in the tumors of serous, endometrioid, and clear-cell ovarian cancers when compared with that in normal ovarian tissues (Figure 3(a) and S1A). Furthermore, we determined their level in the serum specimens using droplet digital PCR to facilitate the possible application in clinical diagnosis. Intriguingly, we observed that only the expression of LINC00909 in the endometrioid, clear-cell, and serous ovarian carcinoma were also substantially elevated when compared with normal ovarian tissues (Figure 3(b)). Moreover, our findings showed that the expression of LINC00909 in the tumor was obviously associated with that in serum with an *R*^2^ of 0.5604 (*p* < 0.001) (Figure 3(c)), implying that the elevation of serum LINC00909 may be the result of high LINC00909 expression in tumors. Altogether, we demonstrated that LINC00909 expression was upregulated in the tumor and serum of patients with ovarian cancer.

Furthermore, we carried out a receiver operating characteristic (ROC) analysis using tumor and serum LINC00909 level (median value as the cutoff) to evaluate their diagnostic performance for OC. Tumor LINC00909 distinguished cancers from normal ovarian tissue with 73.3% of sensitivity and 79.7% of specificity (AUC, 79.0%; 95% confidence interval (95% CI): 73.1%-85.0%) and distinguished serous ovarian cancer from normal ovarian tissue with 82.2% of sensitivity and 79.7% of specificity (AUC, 84.4%; 95% CI: 79.5%-89.3%) (Figure 3(d)). More interesting, serum LINC00909 showed a more considerable diagnostic performance in identifying ovarian cancer (sensitivity, 87.8%; specificity, 69.6%; and AUC, 81.2%) and serous ovarian cancer subtype (sensitivity, 90.0%; specificity, 75.9%; and AUC, 84.5%) from normal ovarian tissues (Figure 3(e)).

Besides, we observed that the tumor and serum LINC00909 in patients with advanced-stage OC were higher than that in FIGO stage I but not stage II (Fig. S1B). In the performance status of patients, our results showed that the ECOG score was positively associated with the tissue and serum LINC00909 level (Fig. S1C). The evidence suggests high LINC00909 level is an unfavorable indicator for patients with OC.

### 3.3. Serum LINC00909 Exhibits Considerable Diagnostic Performance for OC

**Figure 4:**
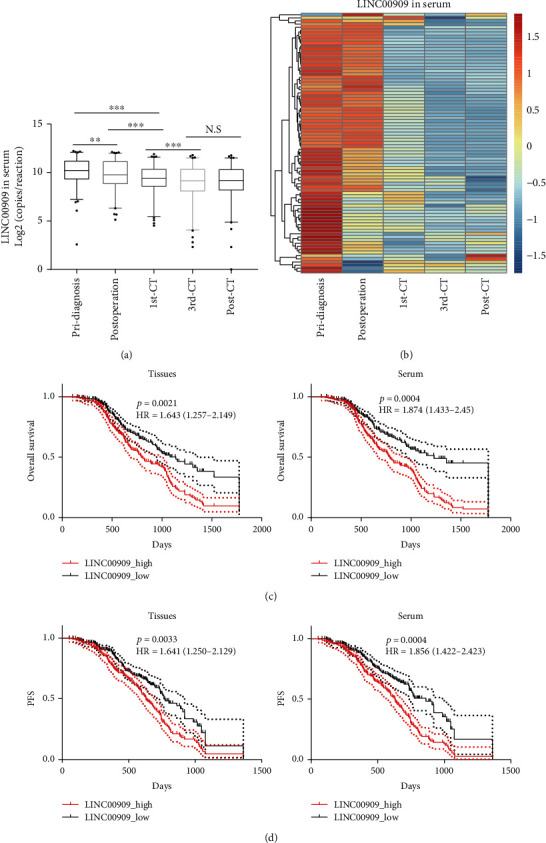
Serum LINC00909 exhibits considerable diagnostic performance for OC. (a, b) The level of lncRNAs was determined by droplet digital PCR. 83 follow-up serum samples were collected at one-week postprimary surgery (postdiagnosis), one-week post the first cycle of platinum-based chemotherapy (1^st^ CT), one-week post 3rd CT (3^rd^ CT), and one-week post the end cycle of CT (post-CT) were used for lncRNA quantification. (c, d) LINC00909-based overall and progression-free survival analyses of OC. HR: hazard ratio. Log-rank (Mantel-Cox) test for statistical analysis.

We also tracked serum LINC00909 levels in follow-up samples (*n* = 83) collected at one-week postprimary surgery, one-week post the first cycle of platinum-based chemotherapy (CT), one-week post 3^rd^ CT, and one-week post the end cycle of CT. We found that, after the primary surgery, the serum LINC00909 level of 43% of patients dramatically reduced to less than 0.5-fold of the initial level (Figures 4(a) and 4(b)). Moreover, the postoperation serum LINC00909 levels were further downregulated after the first and third cycles of CT (Figures 4(a) and 4(b)). However, after the last cycle of CT, the serum LINC00909 level failed to alter when compared with that after the 3^rd^ CT (Figures 4(a) and 4(b)).

Besides, we performed overall and progression-free (PFS) survival analyses using both tissue and serum LINC00909 level (median expression value as the cutoff) of the primary diagnosis. As shown in Figure 4(c), low tissue and serum LINC00909 are favorable for the patients with ovarian cancers, with a hazard ratio of 1.643 (95% CI: 1.257-2.149, *p* = 0.0021) and 1.874 (95% CI: 1.433-2.450, *p* = 0.0004), respectively (Figure 4(c)). Likewise, the patients with low tissue or serum LINC00909 level had longer progression-free survivals with a hazard ratio of 1.471 (95% CI: 1.050-1.929, *p* = 0.0033) and 1.656 (95% CI: 1.022-2.223, *p* = 0.0017), respectively (Figure 4(d)). Altogether, a low (serum) LINC00909 level is favorable for patients with ovarian cancer.

### 3.4. LINC00909 Functions as an Oncogenic Factor to Ovarian Cancer Cells

**Figure 5:**
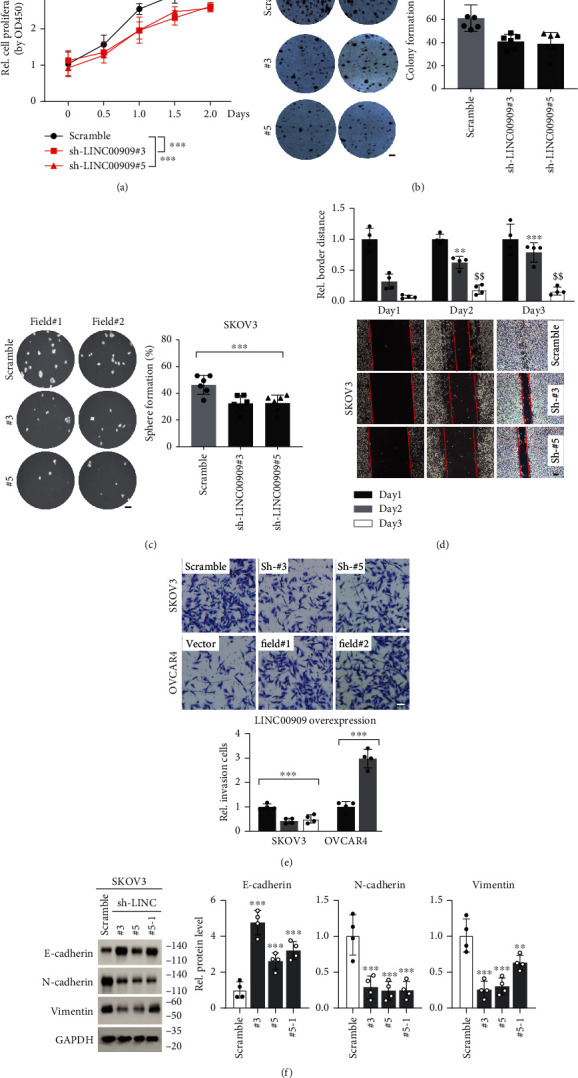
LINC00909 functions as an oncogenic factor to ovarian cancer cells. (a) MTT assay determined cell proliferation of SKOV3 cells. ^#^ indicates independent targets. *N* = 4. (b, c) Colony formation (b) and soft agar (c) assays revealed the tumor characteristics of each SKOV3 cell line. Cells in (a) were used. *N* = 6; scale bar, 1 mm. (d) Wound healing assay determined the migration of SKOV3. Cells in (a) were used. ^∗^Comparisons with day 2 of the scramble group; ^$^comparisons with day 3 of the scramble group; scale bar, 200 *μ*m. (e) Transwell assay determined the invasion of SKOV3 and OVCAR4 cells. The cells were the same with (a) and S2C. Scale bar, 50 *μ*m. (f) Western blot showed the protein level of EMT markers in SKOV3 cells. Cells in (a) were used. E-cadherin, epithelial marker; N-cadherin/vimentin, mesenchymal marker.

To investigate the biological functions of LINC00909 in the tumorigenesis of ovarian cancer, we firstly detected its levels in 6 ovarian cancer cell lines, such as JHOS4, SKOV3, A2780, CAOV4, EFO21, and OVCAR4. Compared with tumor tissues, there was a higher LINC00909 level in JHOS4, SKOV3, and A2780, and in contrast, a lower expression in OVCAR4 (Fig. S2A). Therefore, we knocked down LINC00909 in SKOV3 using an shRNA vector and overexpressed it in OVCAR4 (Fig. S2B and C). Interestingly, knockdown of LINC00909 expression in SKOV3 cells significantly inhibited cell proliferation determined by MTT assay (Figure 5(a)), and in contrast, ectopic LINC00909 efficiently promoted cell growth of OVCAR4 cells (Fig. S2D). Consistently, we observed that knockdown of LINC00909 expression abated about 32.7% colony formation of SKOV3 cells (Figure 5(b)), and ectopic LINC00909 in OVCAR4 cells enhanced colony formation by 56.1% of the vector group (Fig. S2E). In soft agar assay, our results showed that LINC00909 contributed to cells' anchorage-independent growth ability, indicated by the reduction in tumorsphere number of LINC00909-knockdown SKOV3 cells and the increased number in LINC00909-overexpressed OVCAR4 cells (Figure 5(c) and S2F).

In the wound-healing assay, we found that, after 3 days of growth, the scratch of LINC00909-knockdown SKOV3 cells was significantly wider than that of the scramble group (Figure 5(d)), suggesting cells' migration is injured by loss-function of LINC00909. Meanwhile, our data also showed that overexpression of LINC00909 in OVCAR4 cells promoted the healing of scratch (Fig. S2G). Furthermore, we carried out a transwell assay using the cells mentioned above to evaluate their invasive ability. Our results revealed that knockdown of LINC00909 in SKOV3 cells restrained cell invasion, supported by the fewer cell numbers in two independent LINC00909-knockdown cell lines in the transwell assay (Figure 5(e)). In another way, overexpression of LINC00909 in OVCAR4 cells remarkably enhanced cell invasion (Figure 5(e)). Besides, we observed that ectopic LINC00909 in OVCAR4 cells decreased E-cadherin and increased N-cadherin/vimentin expression, demonstrating that it promotes epithelial-to-mesenchymal transition (EMT) of ovarian cancer cells (Fig. S2H). In contrast, knockdown of LINC00909 expression partially reversed the process of EMT in SKOV3 cells (Figure 5(f)). Taken together, LINC00909 contributes to cell proliferation, colony formation, migration, invasion, and EMT of ovarian cancer cells *in vitro*.

### 3.5. miR-23b-3p Is a Critical Functional Mediator of LINC00909

**Figure 6:**
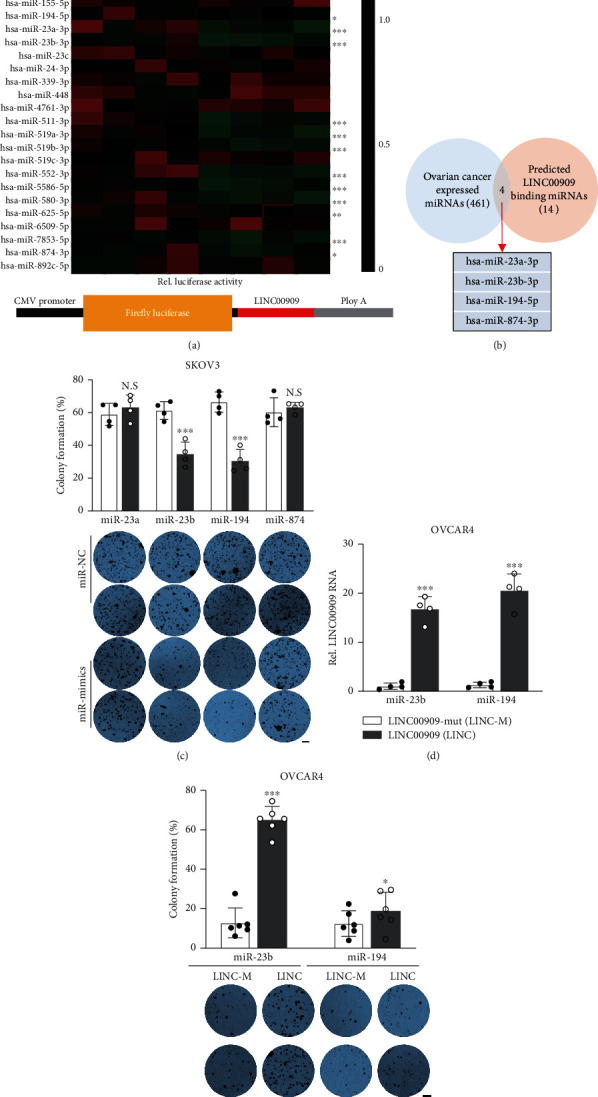
miR-23b-3p is a critical functional mediator of LINC00909. (a) Dual-luciferase reporter assay verified the interaction between miRNAs and LINC00909. 293T cells were transiently cotransfected with LINC00909-Firefly-luciferase construct and miRNA-mimics/negative control. *N* = 4. (b) Venn diagram showed the overlap between ovarian cancer expressed and LINC00909 binding miRNAs. (c) Colony formation assay revealed the tumor characteristics of each SKOV3 cell line. miRNA expression level was presented in S3B. Scale bar, 1 mm. (d) The level of LINC00909 was determined by real-time qPCR. OVCAR4 cells were stably transfected with LINC00909-mut (LINC-m, miRNA binding site mutant) or LINC00909 (LINC). (e) Colony formation assay revealed the tumor characteristics of each OVCAR4 cell line. Cells in S3D were used. Scale bar, 1 mm.

Currently, accumulating reports have indicated that lncRNAs, especially the cytoplasmic ones, involve the pathogenesis of various tumors by serving as competing endogenous RNAs (ceRNAs) to sponge functional miRNAs [32–34]. In the case of LINC00909, the expression and subcellular location data from cell lines showed that it mainly localizes in the cytoplasm (Fig. S3A), showing it is a potential ceRNA. Therefore, we analyzed the 33 putative miRNA interactors of LINC00909 from the STARBASE database (Table S1) and then verified their interaction using dual-luciferase reporter assay in 293T cells. Interestingly, we identified 14 miRNAs, such as miR-105-5p, miR-135b-5p, miR-23a/b, and miR-511-3p, those bound to LINC00909 (Figure 6(a)). In 461 miRNAs that are relatively highly expressed in ovarian cancer, we observed 4 validated LINC00909 binding miRNAs, including miR-23a/b-3p, miR-194-5p, and miR-874-3p (Figure 6(b)). Therefore, we hypothesized that LINC00909 achieves its biofunctions by sponging a certain one(s) of these four miRNAs. Next, we explored the functional roles of these 4 miRNAs in tumorigenesis using colony formation assay. Intriguingly, our results revealed that ectopic miR-23b-3p or miR-194-5p in SKOV3 cells significantly restrained colony formation (Figure 6(c) and S3B), indicating they are tumor suppressors in ovarian cancer *in vitro*. Besides, in miR-23b-3p but not miR-194-5p overexpressed OVCAR4 cells, ectopic LINC00909 still promotes colony formation (Figures 6(d) and 5(e)), suggesting that miR-23b-3p is the downstream functional mediator of LINC00909. Altogether, our results demonstrated that LINC00909 functions as an oncogene in ovarian cancer by sponging miR-23b-3p.

### 3.6. LINC00909 Functions as a ceRNA of MRC2, LASP1, and ZNF839 by Sponging miR-23b-3p

To further investigate the mechanism that miR-23b-3p involves in the pathogenesis of ovarian cancer, we analyzed its targets using three different algorithms from miRcode, TargetScan, and mRDB databases, respectively. We finally identified 20 putative mRNA targets of miR-23b-3p, including MBTD1, STARD3NL, and ZNF839 (Figure 1(a)). To validate if these mRNAs are really the targets of miR-23b-3p, we determined the alteration in their expression level when miR-23b-3p was overexpressed in SKOV3. Intriguingly, our real-time qPCR results showed that the expression of ZNF839, LASP1, DEPDC1, and MRC2 in mRNA level was obviously downregulated in miR-23b-3p overexpressed SKOV3 cells (Figure 1(b)). Consistently, the dual-luciferase reporter assay in 293T cells also indicated that miR-23b-3p directly interacted with the mRNA of these four genes (Figures 1(c) and 1(d)). These observations collectively imply that LINC00909 may function as a ceRNA of ZNF839, LASP1 DEPDNC1, and MRC2 by sponging miR-23b-3p in ovarian cancer cells. Interestingly, we found that, in the ovarian cancer RNA expression datasheet from the GEPIA database, ZNF839, DEPDC1, and MRC2 were associated with LINC00909 in the RNA level (Fig. S4A). Similarly, in the clinical specimens we collected, we also observed that MER2, ZNF839, and LASP1 exhibited a moderated correlation with LINC00909 at the RNA level. Meanwhile, our findings revealed that MRC2, LASP1, and ZNF839 were upregulated in ovarian cancers, especially in the subtype of serous ovarian cancer (Fig. S4B). Besides, our real-time qPCR results suggested that, in LINC00909-overexpressed OVCAR4 cells, the mRNA level of MRC2, ZNF839, and LASP1 were substantially elevated (Figure 1(f)). However, these effects were largely blocked by knocking down miR-23b-3p expression by miRNA inhibitor (Figure 1(f) and S4C). Altogether, our findings demonstrated that LINC00909 is a ceRNA of MRC2, LASP1, and ZNF839 mRNA in the mediation of miR-23b-3p in ovarian cancer cells.

### 3.7. LINC00909 Achieves Its Biological Functions in Ovarian Cancer via Regulating MRC2

**Figure 7:**
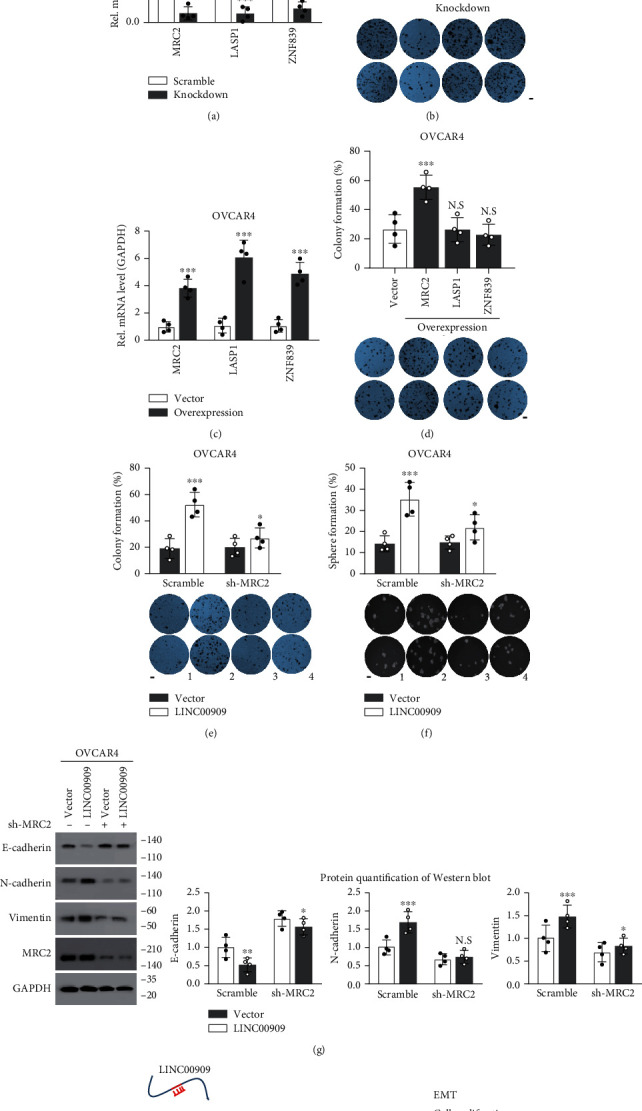
LINC00909 achieves its biological functions in ovarian cancer via regulating MRC2. (a) Real-time qPCR revealed mRNA level of MRC2/ZNF839/DEPC1 in SKOV3 cells. In SKOV3 cells, MRC2/ZNF839/DEPC1 was stably knocked down, respectively, using shRNA vector. Scr: scramble. (b) Colony formation assay revealed the tumor characteristics of each SKOV3 cell line. Cells in (a) were used. (c) Real-time qPCR revealed mRNA level of MRC2/ZNF839/DEPC1 in OVCAR4 cells. In OVCAR4 cells, MRC2/ZNF839/DEPC1 was stably overexpressed, respectively. (d) Colony formation assay revealed the tumor characteristics of each OVCAR4 cell line. Cells in (c) were used. (e, f) Colony formation (e) and soft agar (f) assays revealed the tumor characteristics of each OVCAR4 cell line. Cells in S5A were used. (g) Western blot showed the protein level of EMT markers in OVCAR4 cells. Cells in Figure S5A were used. E-cadherin, epithelial marker; N-cadherin/vimentin, mesenchymal marker. (h) The working model of LINC00909 involves the pathogenesis of ovarian cancer cells.

Furthermore, we knocked down the expression of MRC2, LASP1, and ZNF839 using shRNA construct in SKOV3 cells (Figure 7(a)). Intriguingly, our data showed that only knockdown of MRC2 expression in SKOV3 significantly decreased about 61.3% of colony formation (Figure 7(b)). Meanwhile, we also overexpressed the expression of MRC2, LASP1, and ZNF839 in OVCAR4 cells (Figure 7(c)). Consistently, the colony formation assay results revealed that ectopic MRC2 efficiently increased colony formation in OVCAR4 cells (Figure 7(d)). These findings demonstrated that only MRC2 (but not LASP1 and ZNF839) exhibits oncogenic roles in ovarian cancer cells *in vitro*. To further investigate the relation between LINC00909 and MRC2 in ovarian cancer cells, we overexpressed LINC00909 in MRC2 knockdown OVCAR4 cells (Fig. S5A). In the colony formation assay, we found that the increased colony formation induced by LINC00909 overexpression was obviously blocked after knocking down the expression of MRC2 in OVCAR4 cells (Figure 7(e)). Similarly, downregulation of MRC2 in OVCAR4 cells also largely reduced the tumorsphere formation enhanced by LINC00909 overexpression (Figure 7(f)). These data definitely indicated that MRC2 mediates LINC00909's oncogenic roles in ovarian cancer cells. Previously, MRC2 has been proved to participate in the process of EMT associated with cancer progression by disrupting epithelial cell-cell interactions [35, 36]. In our experiments, we found that the EMT induced by overexpression of LINC00909 in OVCAR4 cells also was remarkably blocked by knockdown of MRC2 expression, indicated by the alteration of E-cadherin/N-cadherin/vimentin protein level in Figure 7(g). Therefore, our observations collectively demonstrated that LINC00909 achieves oncogenic functions in ovarian cancer cells via regulating MRC2.

## 4. Discussion

Over the past decade, a large number of lncRNAs (more than 13000 according to the GENCODE project) have been identified due to the advances in next-generation RNA sequencing and bioinformatics analysis technologies [17–19, 24]. Accumulating studies have demonstrated that lncRNA is implicated in a variety of biological processes and is closely associated with tumor development [13, 15, 18, 19, 27, 37, 38]. LncRNAs also show fine potential in clinical molecular diagnosis in various types of cancers [18, 19]. For example, a clinical investigation by Martini et al. revealed that patients with stage I epithelial ovarian cancer can be clearly stratified into low- and high-risk individuals using the expression profile lncRNAs including PVT1, SERTAD2-3, and miR-200c-3p simultaneously [39].

In the present study, after determining more than 68 lncRNA expression, we found that LINC00909, a long intergenic noncoding RNA, was upregulated in both tumor and serum of patients with OC (including serous, endometrioid, and clear-cell ovarian carcinomas) when compared with that in normal ovarian tissue. Furthermore, the tumor LINC00909 level is positively correlated with that in serum, but whether there is a causality remains further investigation. Moreover, in ROC analysis using LINC00909 level (median as the cutoff), we found the tumor and serum LINC00909 can distinguish OCs from normal ovarian tissues with an AUC of 79.0% (73.1%-85.0%) and 81.2% (76.3%-86.2%), respectively, and show a slightly but significantly better performance in identifying serous ovarian carcinoma from normal ovarian tissues. Also, we demonstrated that the cancer FIGO stages and ECOG score (reflecting patient performance status) positively associated with LINC00909 expression. However, we found that neither tumor nor serum LINC00909 discriminates advanced-stage OC (FIGO stage-III/IV) from early-stage ones (FIGO stage-I/II) (data not shown). The possible explanation is that the number of patients with advanced-stage OC is too small. Furthermore, we noticed that the tumor and serum LINC00909 exhibit a moderate correlation in their expression level, implying the serum LINC00909 may originate from tumor cells. However, our results also showed that quite considerable patients' serum LINC00909 at the point of one-week postprimary surgery significantly elevated when compared to that of pri-diagnosis. After checking the operation efficiency of 83 patients followed up (roughly divided into the total and partial resection groups), we failed to observe a distinguishable difference in serum LINC00909 level between the complete-resection and partial-resection groups (data not shown) at the point of one-week postprimary surgery. We consider that the superficial contradiction may be caused by an unspecific and transient release of LINC00909 into the blood during surgery. Therefore, we suggest that using serum LINC00909 at the point of one-week postprimary surgery as a marker should be avoided in the exploratory investigation for the diagnosis of OC.

Cytoreductive (debulking) surgery and platinum-based chemotherapy are the initial treatment for patients with OC [3, 39]. Different lines of evidence have revealed that lncRNAs are implicated in platinum-resistant ovarian cancers. For example, in recurrent platinum-resistant ovarian tumors, HOTAIR expression was elevated, which contributes to the platinum resistance of OC [40]. In our experiments, we observed that the serum LINC00909 level was downregulated after surgical operation and platinum-based chemotherapy. The phenomenon may be based on two reasons: (1) the serum LINC00909 is derived from ovarian cancer cells (such as tumor-derived exosomes) and (2) interventions cause a substantial reduction in cancer cells. Nevertheless, we failed to observe a distinguishable difference in tumor LINC00909 level between chemotherapy-sensitive and chemotherapy-resistant patients with ovarian cancer. Thus, whether LINC00909 is implicated in the mechanisms of platinum-based chemotherapy resistance needs further investigation.

Furthermore, our results of overall and progression-free (PFS) survival analyses using LINC00909 level showed that low tissue and serum LINC00909 are favorable for the patients with ovarian cancers, corroborated by the relative longer overall and progression-free survivals in patients with low LINC00909 level. In biological functions, we demonstrated that loss-function of LINC00909 in SKOV3 cells (a LINC00909-high ovarian cancer cell line) restrained cell proliferation, migration and invasion, and in contrast, overexpression in OVCAR4 cells (a LINC00909-low ovarian cancer cell line) promoted cell migration and invasion. Likewise, the elevation of LINC00909 expression also was observed in glioma tissues and cell lines, which enhances glioma cell proliferation, invasion *in vitro*, and reduced tumor growth in vivo [41]. More recently, Ma et al. reported that LINC00909 was distinctly upregulated in AML patients and cell lines, and knockdown of LINC00909 suppressed cell viabilities, migration, and invasion [42]. In molecular mechanism, we identified miR-23b-3p as a downstream functional mediator from 33 putative miRNA interactors based on the following findings: (1) dual-luciferase showed that LINC00909 directly interacts with miR-23b-3p, (2) ectopic miR-23b-3p in SKOV3 cells inhibits colony formation, and (3) overexpression of LINC00909 overcomes miR-23b-3p's tumor suppressor effect in OVCAR4 cells. Besides, our observations showed that LINC00909 functions as a ceRNA of MRC2 mRNA by sponging miR-23b-3p. Overexpression of MRC2 in SKOV3 cells efficiently inhibits their colony formation ability. However, LINC00909 in human glioma directly interacts with miR-194 in Liu's study, whereas LINC00909 promotes cell proliferation and metastasis in pediatric acute myeloid leukemia via miR-625-5p [41, 42]. In our results, we also observed that miR-194-5p and miR-625-5p directly interact with LINC00909 in the dual-luciferase assay. Nevertheless, the expression level of miR-625-5p is extremely low in ovarian tissues, which restricts its regulatory potential in the tumorigenesis of ovarian cancer. Although knockdown of miR-194-5p in SKOV3 cells also profoundly inhibited colony formation, overexpression of LINC00909 failed to overcome the effects (colony formation) of ectopic miR-194-5p in SKOV3. Therefore, we consider that miR-23b-3p is a critical functional mediator of LINC00909 in the background of ovarian cancer cells. Meanwhile, the loss-function of MRC2 in OVCAR4 cells also largely blocked the EMT process induced by LINC00909 overexpression. In fact, the elevation of MRC2 protein level has been frequently observed in multiple cancer types, including breast and prostate tumors [36, 43]. Also, MRC2 contributes to the EMT process associated with cancer progression by disrupting epithelial cell-cell interactions [36, 43]. These observations are consistent with our results related to MRC2's functional assay. Therefore, we consider that LINC00909 executes its oncogenic functions, such as promoting cancer cell proliferation, migration, and invasion, by influencing MRC2 expression.

In summary, we demonstrated that the expression of LINC00909 is elevated in the tumor and serum of patients with ovarian cancers including serous, endometrioid, and clear-cell ovarian carcinomas. Serum LINC00909 is effective in diagnosing epithelial ovarian cancers or serous ovarian carcinoma from normal ovarian tissue. High serum LINC00909 level predicts a poor prognosis (including overall and progression-free survival) for OC patients. In mechanism, the elevated LINC00909 level in ovarian cancer cells upregulates the expression of MRC2 by sponging miR-23b-3p, and thereby promotes cell proliferation, migration, invasion, and EMT of ovarian cancer cells (Figure 7(h)). Therefore, we highlight that the LINC00909/miR-23b-3p/MRC2 axis is implicated in the pathogenesis of ovarian cancer, and serum LINC00909 may be a promising biomarker for the diagnosis of OC.

## Figures and Tables

**Table 1:** Characteristics of patients with ovarian cancers.

Characteristics	Number (*N* = 217)
Age	Median (67 years) with range of 21-90 years
FIGO stage	
I	31 (14.3%)
II	33 (15.2%)
III	80 (36.9%)
IV	71 (32.7%)
ECOG	
0	35 (16.1%)
I	54 (24.9%)
II	51 (23.5%)
III	69 (31.8%)
IV	7 (3.2%)
Histologic subtypes	
Serous	170 (78.3%)
Endometriods	25 (11.5%)
Clear cell	21 (9.7%)
Recurrence	
PFS	Median (479 days) with range of 53-1362 days
NO relapse	76 (35%)
Relapse	140 (64.5%)
Survival	
OS	Median (618 days) with range of 102-1774 days
Alive	172 (79.3%)
Dead	45 (20.7%)

FIGO stage (I-IV), FIGO Committee on Gynecologic Oncology 2009 I: tumor confined to ovaries or fallopian tube(s); II: tumor involves one or both ovaries or fallopian tubes with pelvic extension (below pelvic brim) or peritoneal cancer (Tp); III: tumor involves one or both ovaries, or fallopian tubes, or primary peritoneal cancer, with cytologically or histologically confirmed spread to the peritoneum outside the pelvis and/or metastasis to the retroperitoneal lymph nodes; IV: distant metastasis excluding peritoneal metastases. ECOG (0-IV), Eastern Cooperative Oncology Group score 0: fully active, able to carry on all predisease performance without restriction; I: restricted in physically strenuous activity but ambulatory and able to carry out work of a light or sedentary nature, e.g., light housework and office work; II: ambulatory and capable of all self-care but unable to carry out any work activities; up and about more than 50% of waking hours; III: capable of only limited self-care, confined to bed or chair more than 50% of waking hours; IV: completely disabled; cannot carry out any self-care; totally confined to bed or chair; V: dead. PFS: progression-free survival; OV: overall survival.

**Table 2:** Oligos involved in this study.

Gene symbols	For real-time qPCR	
	Sequences	
	Forward (5′ →3′)	Reverse (5′ →3′)
ADIPOQ-AS1	GGATTCCCGGAAAGCCTCGG	GAGATGGCACCCCGTGGACC
AGBL5-AS1	GCGCATTATCGGGCTGTGCC	CACCCGGGACACCTAGACAT
C14orf132	GCCCAGAGACAGCCGAGTGC	AAGTCCTCGTTGGGCGAGTC
CPEB1-AS1	CAAGGGAGCGGGTGTCTGGA	CAGGCAGCTGGCAGGACCTG
DDX11-AS1	GGTTCTGGGAGATGACCAATGG	CCATAGTTTCAGGATGCTGGCAG
ELF3-AS1	CATCACCTGCCTGGACTCCA	GCGGTTCGTGATGACTTCACC
ELOA-AS1	CAGCAAGACTGTCAGCTCGG	AGCCCAGGATACAGAGCCAG
EMX2OS	GCGGAGTATTGATCGGCTGC	GGCATGCCCTGCCTTCATTC
FAM87B	GAAGGCGCCTCCCTCTGTGG	CTGCAGGCCGAGCACACACC
FGD5-AS1	CTCCACCCTGCCTTCTCGCT	CTTCTTAAGCCACCACTGCACTG
HCG23	AGGGCCTTCACAAGCACAGAC	ACTTCTCATGTGTTAGGGAGGCTG
HHIP-AS1	CTTCCCCTCCGCACTGCCTG	CACCACTCTGTCGGTTTAGCTG
HOTAIRM1	AGCAAAAGCTGCGTTCTGCG	AGCTGTTGATGGGTTCAGGCA
HOXA-AS2	CCTGGTGCAAAGTCCCACGC	TTCTCCAAGCCAGGGTCCGG
LIF-AS1	GGCACAGGAGCTGACACTTACA	CCCTTGATCCCAGGAGGTGG
LINC00028	GTCTTCCCCCACCCCAAGCT	GAGACCCAGTGGCTCATGCC
LINC00337	GGGCAGACACAGGGTCTCACT	AGGTCACAAAGCTAGGCCGC
LINC00346	AGTGGACAGTCTCAGGTCAGGG	AGTGGACAGTCTCAGGTCAGGG
LINC00461	TCCCCAGAAGGGAAGACCCAT	CATTTGATCTCCCTGCGCCAGG
LINC00467	CAGCACCGATCCCGACATAGAT	AGGGTCAGCCCAGTTTCAGTC
LINC00484	GCTCCACGGCTCTTGACAGG	TCAGGATCTCAGGTGCGCTG
LINC00515	GTTGAACGGAGCAGTGATGTGG	GCGTGGAGGTCAGGACTACGGA
LINC00852	GGCTAAGGTGGGCATGACCG	AGACAGCACTGAAAGAGCTGCA
LINC00893	CAGGCTGTGCTCACACCAAT	GTGACCACGCAGTAATGCAGC
LINC00908	TGCAGAGTGGGGTCTCCCTG	AGAAACTCCGTCAGTGCTCCC
LINC00909	GCTTGAACCCAGTGGCGGAG	CGCAGCTTAGCTCCCACTTACG
LINC00917	TCCCTAGGCCAGGCTGGAGG	GGGTATGCCCTGTGTGACACC
LINC00921	GCTTGGAAACCCAGCTAGATGG	TCTCCTAACTACTTCCTGCAGCA
LINC00996	GAAGCTGGGAGGAGTCCGCC	GGTCTTGATTGATTCCTGTGTCCC
LINC01039	AGCATGGCCCTTGATCTCAGG	TCAGAGATAGCCACACTGGCC
LINC01088	TGGATTCCCGGAAAGCCTCG	GCCGTGATGGCAGAGATGGC
LINC01140	TTGCTCCCCATCGGTCGCTG	ATCTGTGGGTGACACTGGACC
LINC01197	GTGCTAGCGGTGCCCTTGTG	CAGCTTGGATACCGCTTCTTGG
LINC01320	AGCCGACATCAGTCTCCAGAG	TACCTTCCGCAGATTAGTTACATG
LINC01354	GCCGAGAACACGTCTGGAGC	TCTCAGCAATTTGGGAGACCG
LINC01365	GCTTCCTTGCAAGAACCTTCCC	GAATTAGCCACTTGGCTCTGGC
LINC01391	GGCAGGTAGCAGCCCACCTC	CGCGCAGTGTGGCTACCGAA
LINC01563	GAGAGCAAGCCGGAAGTGCT	CCCCTCAGTCACCGTATTCAG
LINC01626	CTCCCCATCATCCCAGTGTG	AGAGTGGAGTGATTCCTAGCACC
LINC01638	GCTCACCTCCTCCATCCCATG	GCTGGATGTGGTGGTGTGTAAC
LINC01881	CAGTCACAGCATAGGCATGGC	GTGCTCAGCTGAGTCTGCAG
LINC02158	ACACTCACCGCGAGGGTCTG	CAGCGCCTCTGTCACTTTCAG
LINC02278	GATGTTACCCACCACCACCTCT	TGGGGATTAAGCACTCCATCCC
LINC02432	TCATGCCAGGAGAAGCCTCC	ATCAATGCTGGTGGTTGAGGTG
LINC02502	CTCTACTGCCTTCCAAGGTTTC	CCCTGGAATCCAGGACAGCC
LINC02609	TAAGACACCTGCGGAGCTCG	TGCCGTGCTGTCCTCAGCAG
LMF1-AS1	CCCCATCACAGACTGCGCTT	TCTGCCCCGAACTCTCTGGC
MCF2L-AS1	CTCTCTGAGCTCTCCTCGCC	CTTGCTACATTGCCCAGGCTC
MIR100HG	CCCCTCCTCAGGAATGTCTCC	GTTCTAGTGGCAGAAGCTGCAC
MIR3936HG	TCCACCTGCCTCAGCCTCCC	CTGGGGAGGAGGTGTGCTTC
MIR99AHG	GGCAACATAACCAAATGAGCCTTC	CTTCCCATTCTGACCTCAGC
MLIP-AS1	GAAGCTCAGTCTAAGTGCCTGC	ACTTGAGATTAGCTGCAGAGCC
PCAT19	GAGCTCCTCCCTTTCCTCGG	CTTTCCACAGGTGAGGGACC
PCOLCE-AS1	CCTCAAGACAGAGGCAGCAGC	TGCAGGACCGCACGGAGAAG
PGM5-AS1	TGTGGGGACTGTGGGCAGTG	CCCAGCAGGTTTCAACAGACG
PROSER2-AS1	CTGAGTTCAAGCGGTCCTCC	AGCAACCAGCCAGGCAGCAC
RBMS3-AS3	CTACCCCACACAAGAGCTGC	AGGAGTTCCGGAATCCACACC
SEMA3B-AS1	CACAGGTTGGAGGTGGGAGG	CTTCAGGGCTCCACTCTGCC
SLC16A1-AS1	TCCTGAGAGGTCCCATGTGC	CATGTGCCATGTTGGTGTGC
SNAI3-AS1	GATCAGGGTGGCTGTTCTTGG	CCTCGTGGTCACAGCTCAGC
STAM-AS1	CGTGTCCCTCGGAGAGGCTG	GGGTTGCTAGGAATCACTCACTG
SVIL-AS1	TGTTGCACCCTGAAGAGCCTC	GTCTTAGGGACTCAGGGCACC
TRAF3IP2-AS1	CTCACACTTGCCTCGCCGAC	ATCTCAGCAGAATGCTCGTGGG
TTN-AS1	AGAGGCAGGACTGGCTAGGT	AAGAACTGCCAGAGGAGCCAT
TUG1	CATCTCACAAGGCTGCACCAG	CTTAGGTGTCACCACGGTGGT
XIST	ATGTCAAAAGATCGGCCCAGC	ATTAGCTGGAGCTTGGCCAG
ZBTB20-AS5	AGGGAAGGGGAACCTGCTAG	GGGACTTCTCTGTGCTCTGCC
ZEB1-AS1	GAAAGGGACGCCTGGTTTCC	CCACCTAGGATCCCACGGTTC
MBTD1	GGCATGGCTACCTGTGAGATG	GGCCAAAATGCTTGCCTTCT
STARD3NL	TTGGTGGGCAATAGCGTTGA	GCAGCACATAGCCAAAAGCC
ZNF839	GTACAGCCGCTTGTGAGAAC	CTGGGACTCTCTTTGCAGGG
JARID2	ACCAGTCTAAGGGATTAGGACC	TGCTGGGACTATTCGGCTGA
NRXN3	AGTGGTGGGCTTATCCTCTAC	CCCTGTTCTATGTGAAGCTGGA
FAS	TCTGGTTCTTACGTCTGTTGC	CTGTGCAGTCCCTAGCTTTCC
CALCR	TTACCCGCATACCAAGGAGAA	TGGGCAGAACTGATAGGACAATA
LASP1	CGAGAAGAAGCCCTACTGCAA	CTGCCACTACGCTGAAACCT
DEPDC1	TTTTGGTCCTGAAGTTACAAGGC	TGGATACCTTCGTGGTAGAGTTT
SS18L2	ATCCGCTGTATTGTGGAGTATCA	GCATCTGCAATGGTAGCCAAAT
CREBBP	CAACCCCAAAAGAGCCAAACT	CCTCGTAGAAGCTCCGACAGT
MRC2	CGAGGAGGACCTATGTGCTCT	CGCTCGTCTTTGCCGTAGT
PIK3C2A	AAATGGGACCAGTAGTTTGCC	GGGTTTGTGCGGTGATTGGTA
ALAS1	AGGCCAAGGTCCAACAGACT	TCCTCACGGCATTCATTTCCT
ETV1	TGGCAGTTTTTGGTAGCTCTTC	CGGAGTGAACGGCTAAGTTTATC
BTBD7	AAAGGAGCTTTCTCTACAAGCC	GCCCCATACTCTGGTGAGGAA
MAP3K9	TACCCGCCCATTCAGTTGTTA	CCTCTTGGCGAACATTCTCTATG
HOXA11	TGCCAAGTTGTACTTACTACGTC	GTTGGAGGAGTAGGAGTATGTCA
ISL1	GCGGAGTGTAATCAGTATTTGGA	GCATTTGATCCCGTACAACCT
	ShRNA sequences (5'- >3')	
LINC00909#1	CCGGGAGGGCTGTCTGGGATTTCTACTCGAGTAGAAATCCCAGACAGCCCTCTTTTTTG	
	AATTCAAAAAAGAGGGCTGTCTGGGATTTCTACTCGAGTAGAAATCCCAGACAGCCCTC	
LINC00909#2	CCGGGCAAGGCGAGAATGCTACTTTCTCGAGAAAGTAGCATTCTCGCCTTGCTTTTTTG	
	AATTCAAAAAAGCAAGGCGAGAATGCTACTTTCTCGAGAAAGTAGCATTCTCGCCTTGC	
LINC00909#3	CCGGCCGATTTCGAGAAATGACTTTCTCGAGAAAGTCATTTCTCGAAATCGGTTTTTTG	
	AATTCAAAAAACCGATTTCGAGAAATGACTTTCTCGAGAAAGTCATTTCTCGAAATCGG	
LINC00909#4	CCGGGACTCTGTAATGGGAAGTTATCTCGAGATAACTTCCCATTACAGAGTCTTTTTTG	
	AATTCAAAAAAGACTCTGTAATGGGAAGTTATCTCGAGATAACTTCCCATTACAGAGTC	
LINC00909#5	CCGGCTTGCTAAAGAGGTTTGACTACTCGAGTAGTCAAACCTCTTTAGCAAGTTTTTTG	
	AATTCAAAAAACTTGCTAAAGAGGTTTGACTACTCGAGTAGTCAAACCTCTTTAGCAAG	
MRC2#1	CCGGCCGGTATTGCTATAAGGTGTTCTCGAGAACACCTTATAGCAATACCGGTTTTTG	
	AATTCAAAAACCGGTATTGCTATAAGGTGTTCTCGAGAACACCTTATAGCAATACCGG	
MRC2#2	CCGGGAAATGAATGAGCAGCAAGAACTCGAGTTCTTGCTGCTCATTCATTTCTTTTTG	
	AATTCAAAAAGAAATGAATGAGCAGCAAGAACTCGAGTTCTTGCTGCTCATTCATTTC	
LASP1#1	CCGGCCAGGACCAGATCAGTAATATCTCGAGATATTACTGATCTGGTCCTGGTTTTTG	
	AATTCAAAAACCAGGACCAGATCAGTAATATCTCGAGATATTACTGATCTGGTCCTGG	
LASP1#2	CCGGGAACTACAAGGGCTACGAGAACTCGAGTTCTCGTAGCCCTTGTAGTTCTTTTTG	
	AATTCAAAAAGAACTACAAGGGCTACGAGAACTCGAGTTCTCGTAGCCCTTGTAGTTC	
ZNF839#1	CCGGGACTTCCGATGGGCTTATCTTCTCGAGAAGATAAGCCCATCGGAAGTCTTTTTTG	
	AATTCAAAAAAGACTTCCGATGGGCTTATCTTCTCGAGAAGATAAGCCCATCGGAAGTC	
ZNF839#2	CCGGGAGCCATGAGTTACTGTCTCACTCGAGTGAGACAGTAACTCATGGCTCTTTTTTG	
	AATTCAAAAAAGAGCCATGAGTTACTGTCTCACTCGAGTGAGACAGTAACTCATGGCTC	

## Data Availability

The datasets generated during and/or analyzed during the current study are available from the corresponding author on reasonable request.

## Supplementary Materials

Supplementary Materials Figure S1: LINC00909 expression is elevated in tumors and serum of OC patients. (a) The level of lncRNAs was determined by real-time qPCR. Tissues of normal ovarian (*N* = 89), serous (*N* = 170), endometrioid (*N* = 25), and 22 clear-cell ovarian cancer (*N* = 22) were used for lncRNA relative quantification. (b) The level of serum LINC00909 among different FIGO stages was determined by droplet digital PCR. Tissues of normal ovarian (*N* = 89), serous (*N* = 170), endometrioid (*N* = 25), and 22 clear-cell ovarian cancer (*N* = 22) were used for lncRNAs quantification. (c) The level of serum LINC00909 among patients with different ECOG scores was determined by droplet digital PCR. Tissues of normal ovarian (*N* = 89), serous (*N* = 170), endometrioid (*N* = 25), and 22 clear-cell ovarian cancer (*N* = 22) were used for lncRNA quantification. Figure S2: LINC00909 is essential for the migration and ovarian carcinoma cells. (A–C)The level of LINC00909 was determined by real-time qPCR. In (B), ^#^ indicates SKOV3 cell lines which were stably transfected with independent shRNA vector. In (C), LINC00909 was stably transfected into OVCAR4 cells (*N* = 4). (a) MTT assay determined cell proliferation of OVCAR4 cells. Cells in S2C were used. (E, F) Colony formation (E) and soft agar (F) assays revealed the tumor characteristics of each OVCAR4 cell line. Cells in S2C were used. (G) Wound-healing assay determined the migration of OVCAR4. Cells in S2C were used. ^∗^Comparisons with day 2 of scramble group; ^$^comparisons with day 3 of the scramble group. (H) Western blot showed the protein level of EMT markers in OVCAR4 cells. Cells in S2C were used. E-cadherin, epithelial marker; N-cadherin/Vimentin, mesenchymal marker. Figure S3: miR-23b is a critical functional mediator of LINC00909. (a) The expression and subcellular location of LINC00909 data in different cell lines are from the lncATLAS database. DANCR serves as a cytoplasmic lncRNA marker; MEAT as a nuclear lncRNA marker. Relative concentration index (RCI). C: cytoplasmic; N: nuclear. (b) The level of indicated miRNAs was determined by real-time qPCR. SKOV3 cells were stably transfected with the indicated miRNAs. Figure S4: LINC00909 functions as a ceRNA by sponging miR-23b in ovarian cancer cells. (a) Scatter plot showed the correlation between LINC00909 and MRC2/ZNF839/DEPC1/LASP1 mRNA. GAPDH serves as a negative control. Data were collected from the GEPIA database. *R*: Pearson correlation coefficient. (b) The mRNA level of indicated genes was determined by real-time qPCR. Tissues of normal ovarian (*N* = 89), serous (*N* = 170), endometrioid (*N* = 25), and 22 cell ovarian cancer (*N* = 22) were used for expression level quantification. (c) The level of miR-23b-3p in OVCAR4 cells was determined by real-time qPCR. LINC00909 was stably transfected, and miR-23b-3p inhibitor was transiently transfected. Figure S5: LINC00909 achieves its biological functions in ovarian cancer via regulating MRC2. (a) The MRC2/LINC00909 level in OVCAR4 cells was determined by real-time qPCR. In OVCAR4 cells, LINC00909 was stably overexpressed, and MRC2 was stably knocked down using an shRNA vector.
