# Supplementary material for: Elevated LINC00909 Promotes Tumor Progression of Ovarian Cancer via Regulating the miR-23b-3p/MRC2 Axis
Source: Oxid Med Cell Longev. 2021 Jul 21;2021:5574130. doi: 10.1155/2021/5574130 (PMC8318762; doi:10.1155/2021/5574130)

Fig. S1 LINC00909 expression is elevated in tumors and serum of OV patients

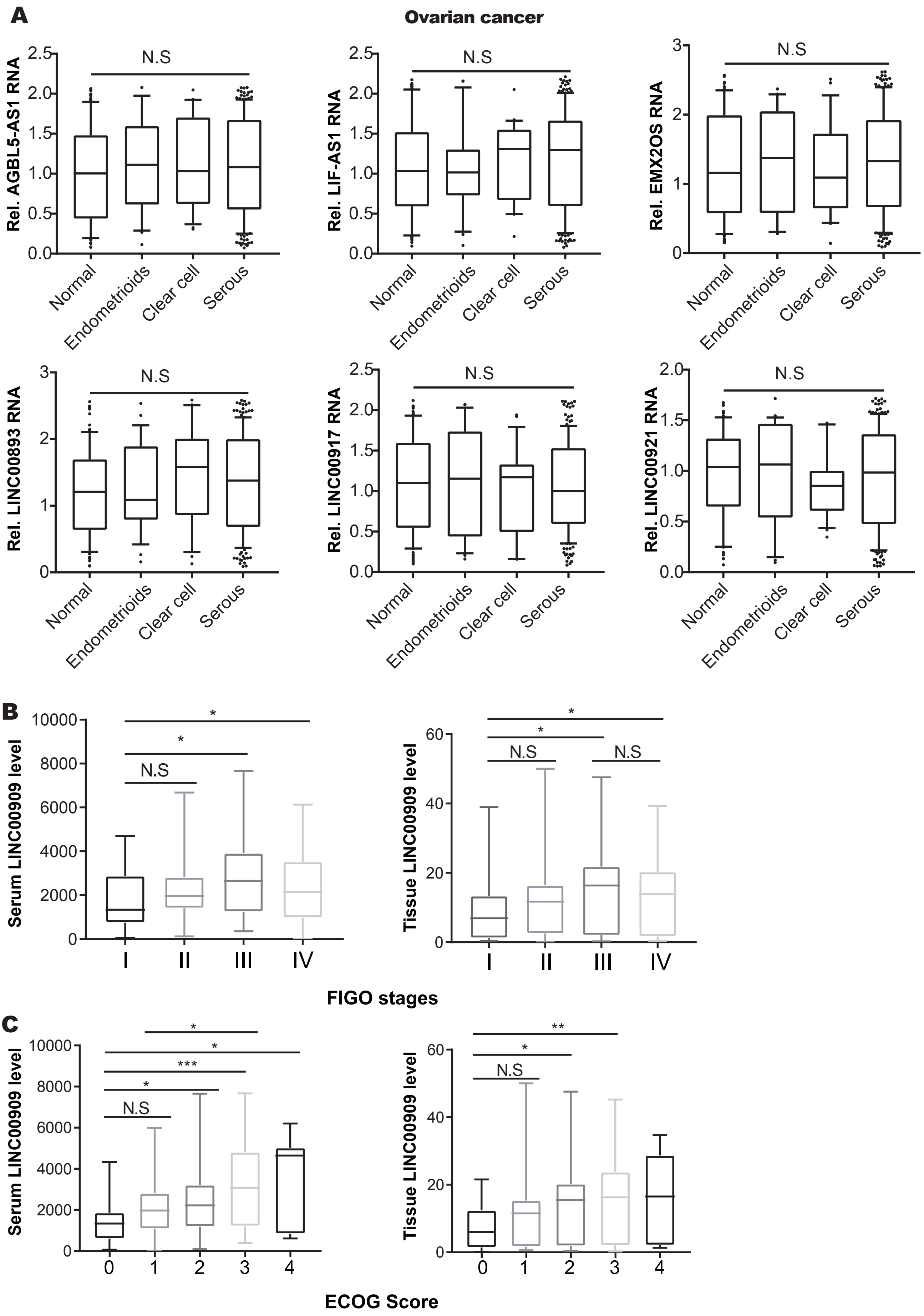

Fig. S2 LINC00909 is essential for the migration of ovarian carcinoma cells

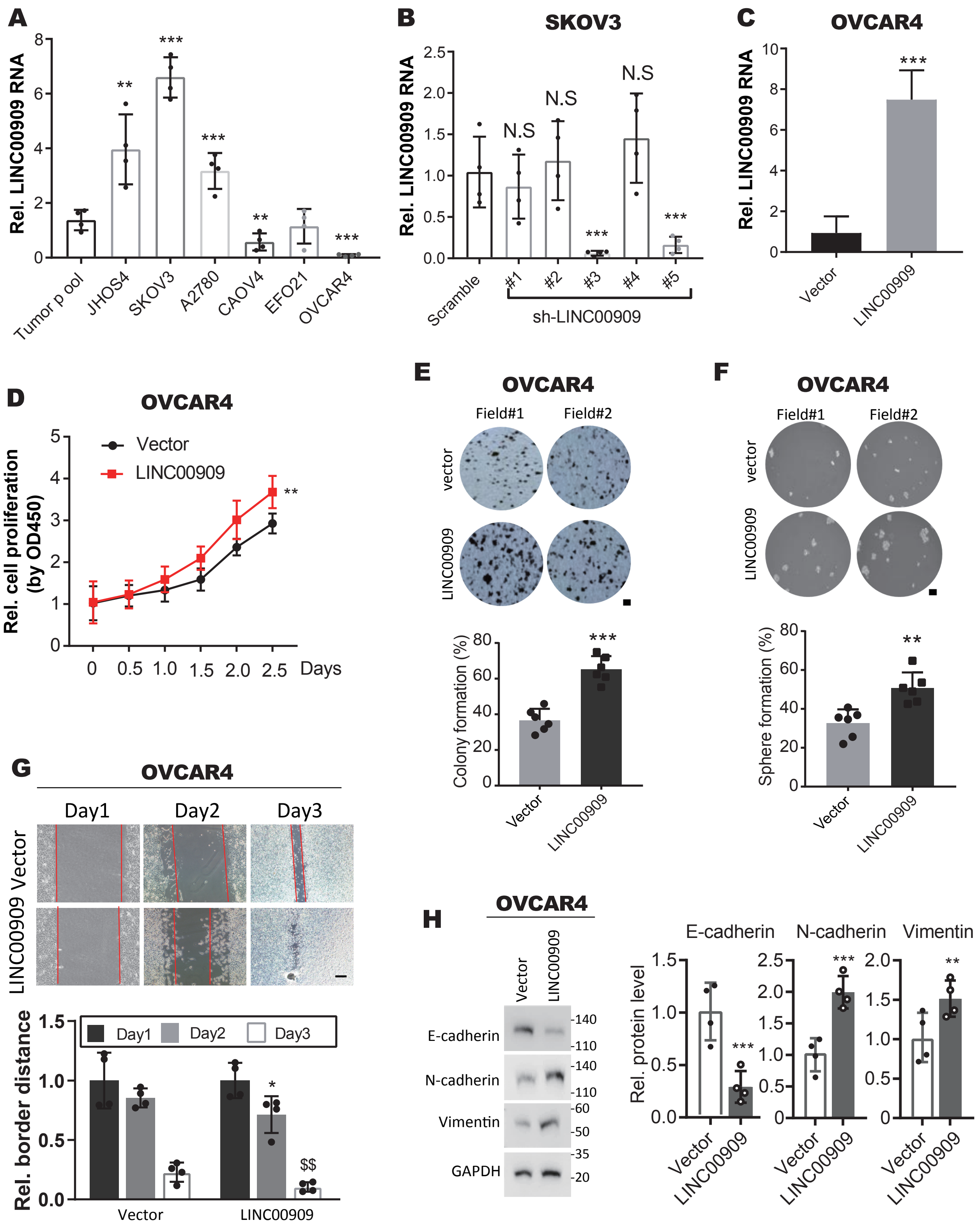

Fig. S3 miR-23b is a critical functional mediator of LINC00909

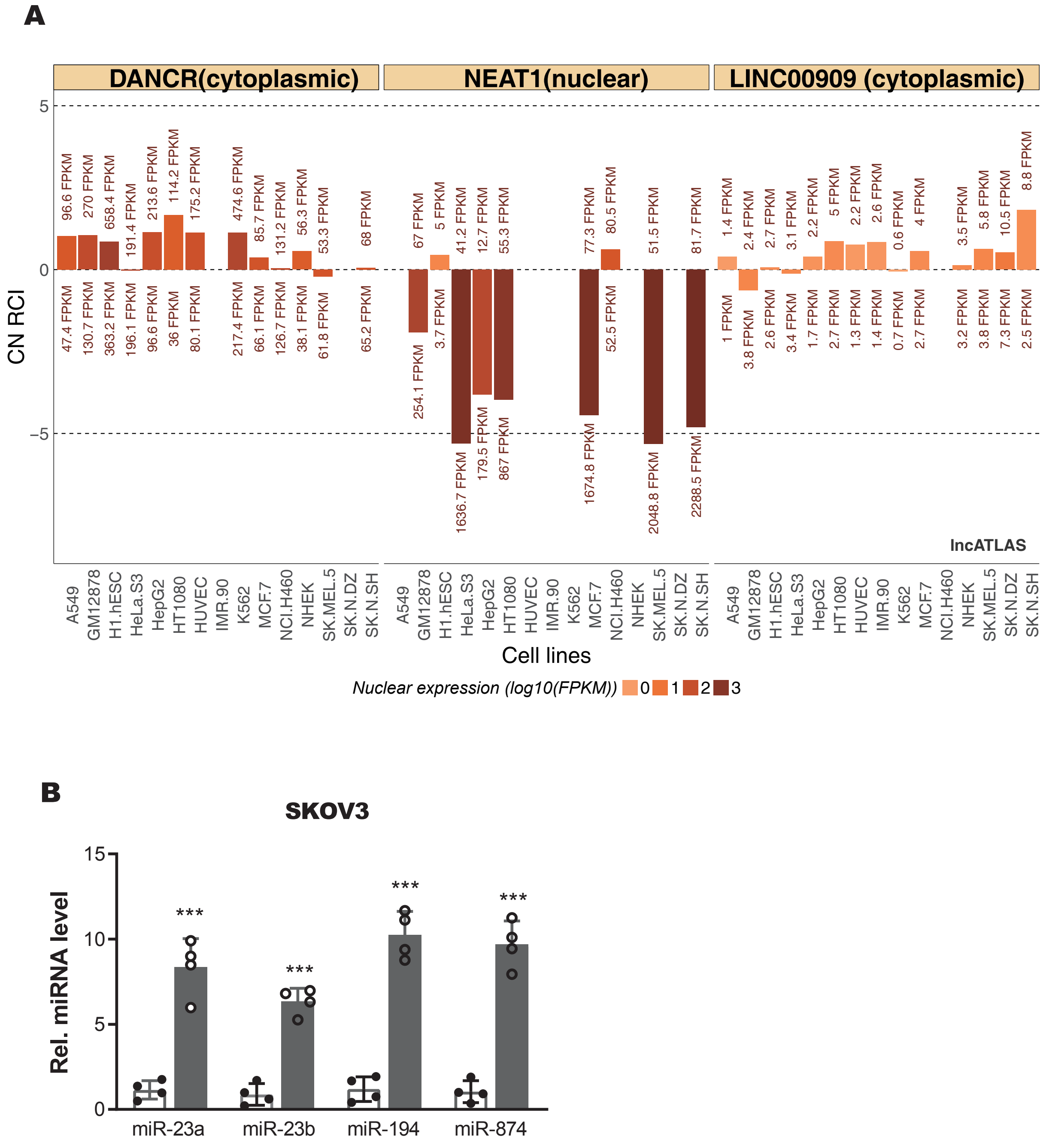

Fig. S4 LINC00909 serves as a ceRNA by sponging miR-23b in ovarian cancer cells

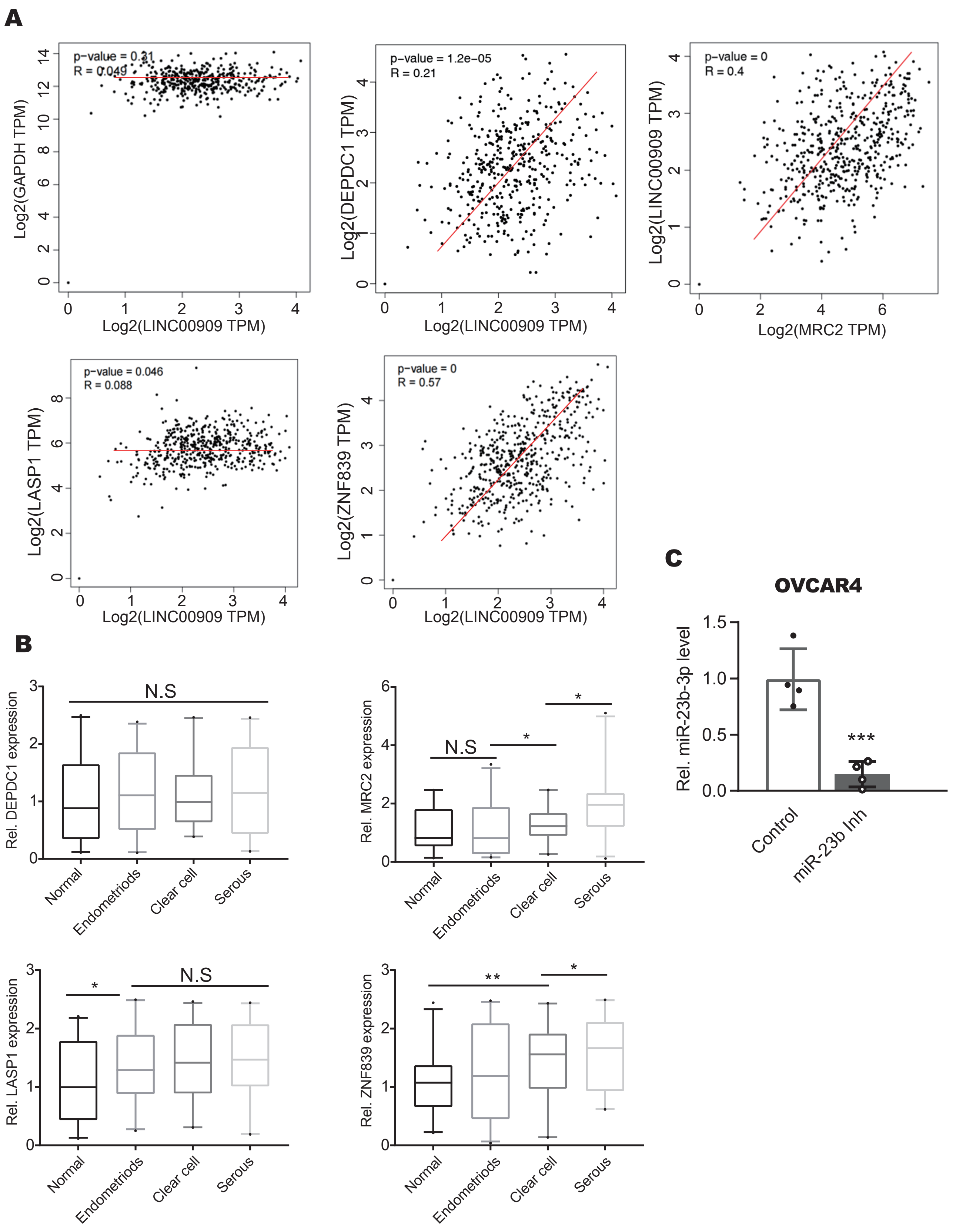

Fig. S5 LINC00909 achieves its biological functions in ovarian cancer via regulating MRC2

A

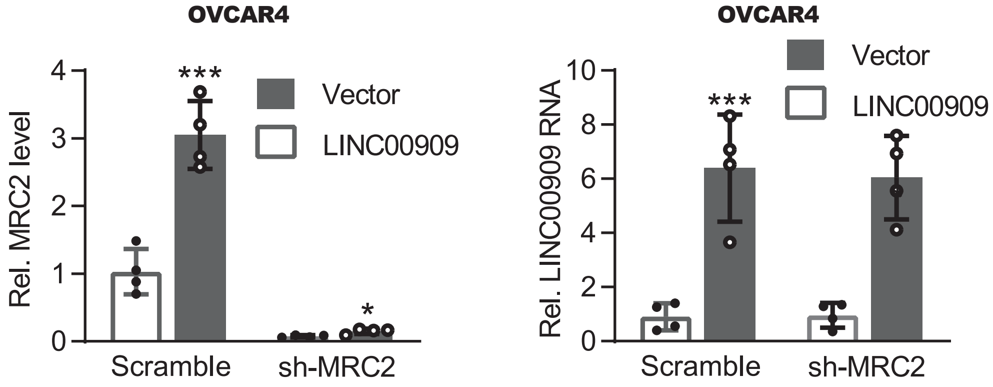

Supplement: Supplementary Materials — Figure S1: LINC00909 expression is elevated in tumors and serum of OC patients. (a) The level of lncRNAs was determined by real-time qPCR. Tissues of normal ovarian (N = 89), serous (N = 170), endometrioid (N = 25), and 22 clear-cell ovarian cancer (N = 22) were used for lncRNA relative quantification. (b) The level of serum LINC00909 among different FIGO stages was determined by droplet digital PCR. Tissues of normal ovarian (N = 89), serous (N = 170), endometrioid (N = 25), and 22 clear-cell ovarian cancer (N = 22) were used for lncRNAs quantification. (c) The level of serum LINC00909 among patients with different ECOG scores was determined by droplet digital PCR. Tissues of normal ovarian (N = 89), serous (N = 170), endometrioid (N = 25), and 22 clear-cell ovarian cancer (N = 22) were used for lncRNA quantification. Figure S2: LINC00909 is essential for the migration and ovarian carcinoma cells. (A–C)The level of LINC00909 was determined by real-time qPCR. In (B), # indicates SKOV3 cell lines which were stably transfected with independent shRNA vector. In (C), LINC00909 was stably transfected into OVCAR4 cells (N = 4). (a) MTT assay determined cell proliferation of OVCAR4 cells. Cells in S2C were used. (E, F) Colony formation (E) and soft agar (F) assays revealed the tumor characteristics of each OVCAR4 cell line. Cells in S2C were used. (G) Wound-healing assay determined the migration of OVCAR4. Cells in S2C were used. ∗Comparisons with day 2 of scramble group; $comparisons with day 3 of the scramble group. (H) Western blot showed the protein level of EMT markers in OVCAR4 cells. Cells in S2C were used. E-cadherin, epithelial marker; N-cadherin/Vimentin, mesenchymal marker. Figure S3: miR-23b is a critical functional mediator of LINC00909. (a) The expression and subcellular location of LINC00909 data in different cell lines are from the lncATLAS database. DANCR serves as a cytoplasmic lncRNA marker; MEAT as a nuclear lncRNA marker. Relative concentration index ( [file 5574130.f1.pdf]
